# 

**Published:** 1893-07

**Authors:** 


ESTABLISHED 1855. SUBSCRIPTION, $2.00.

LIBRARY Indexed S. G. O.

### ATLANTA MEDICAL AND SURGICAL JOURNAL.

OLD SERIES, VOL. XXVI. NEW SERIES, VOL. X. | ATLANTA, GA., JULY, 1893. No. 5.

LUTHER B. GRANDY, M. D., MANAGING EDITOR. M. B. HUTCHINS, M. D., BUSINESS MANAGER.

